# A practical CO_2_-mediated synthesis of 5,6-carboxylated silicon-rhodamines for targeted probe development

**DOI:** 10.3762/bjoc.22.72

**Published:** 2026-06-10

**Authors:** Dongjie Hou, Shaowei Wu, Ning Xu, Pengjun Bao, Wenhao Jia, Qinglong Qiao, Zhaochao Xu

**Affiliations:** 1 State Key Laboratory of Chemical Reaction Dynamics, Dalian Institute of Chemical Physics, Chinese Academy of Sciences, Dalian 116023, China; 2 University of Chinese Academy of Sciences, Beijing 100049, China

**Keywords:** carboxylation, CO_2_, fluorescent probes, silicon-rhodamine, super-resolution imaging

## Abstract

Silicon-rhodamine (SiR) dyes are among the most important fluorophores for super-resolution imaging owing to their far-red emission, high photostability, and tunable lactone–zwitterion equilibrium. However, their broader application in targeted probe development has been limited by the challenging synthesis of 5,6-carboxylated SiR derivatives, which are essential intermediates for bioconjugation. Here, we report a practical CO_2_-mediated strategy for the synthesis of 5,6-carboxylated SiRs from brominated SiR precursors via lithium–halogen exchange and direct carboxylation. Using *n*-BuLi and readily available CO_2_, this method delivers carboxylated SiR derivatives in 60–93% yields while avoiding the use of *t*-BuLi, toxic CO, and expensive palladium catalysts. In addition, the crude carboxylation mixtures can be directly subjected to amide coupling without chromatographic purification, enabling one-pot access to HaloTag-targeted SiR probes. The resulting probes were successfully applied to long-term live-cell super-resolution imaging, allowing visualization of filopodial dynamics and mitochondrial remodeling. This work establishes a concise and efficient route to functionalized SiR dyes and facilitates the rapid development of targeted SiR probes.

## Introduction

**Figure 1:**
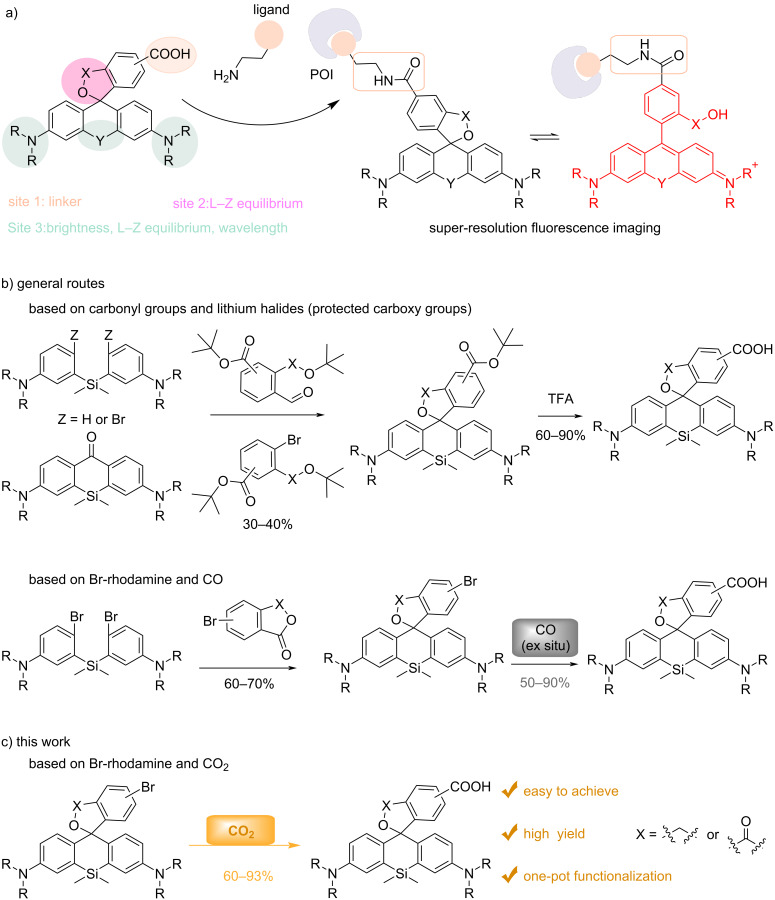
(a) Modular structure of spirocyclic rhodamines. (b) Conventional carboxylation strategies. (c) CO_2_-based direct carboxylation method developed in this work.

Rhodamine dyes, which enable fluorescence imaging in living cells – a powerful technique for studying biological systems in vivo [1–2] – have emerged as one of the most important fluorophores for live-cell bioimaging [3–7] by virtue of their high brightness, excellent photostability [8–10], and, most importantly, tunable lactone–zwitterion (L–Z) equilibrium [11–13]. This distinctive feature enables controllable fluorescence switching and fluorogenic responses, making rhodamines particularly valuable for advanced imaging beyond the optical diffraction limit [14–19]. Replacement of the conventional bridging oxygen atom with silicon shifts the emission of rhodamines from red to the far-red region (typically λ_em_ ≈ 650–670 nm) while retaining high brightness and excellent photostability [20–24]. As a result, silicon-rhodamines (SiRs) have become the most practical rhodamine scaffolds for live-cell imaging, benefiting from reduced cellular autofluorescence and phototoxicity, together with favorable cell permeability [25–26]. Johnsson and co-workers further showed that SiRs can exist in a nonfluorescent spirolactone form, whereas target binding shifts the equilibrium toward the fluorescent zwitterionic form, thereby generating a strong fluorogenic response (Figure 1a) [27–29]. This behavior makes SiRs highly attractive as fluorogenic probes for precise super-resolution imaging by enabling target-specific recognition and significantly improving signal-to-noise ratio (SNR). In addition, hydroxymethyl-substituted SiR derivatives, including **HMSiR** [30–31] and **Aze-HMSiR** [32–34], can undergo spontaneous blinking and have become indispensable tools for live-cell single-molecule localization microscopy (SMLM), highlighting the versatility of SiR dyes in live-cell imaging [35–36].

Building on this foundation, structural modification of SiR dyes has evolved into a well-established molecular design framework (Figure 1a). Conformational restriction and optimization of amino substituents have been widely employed to enhance brightness and photostability [8,37–39], whereas extension of π-conjugation and electronic tuning have expanded the emission of SiR dyes from the far-red to the near-infrared region, reaching up to 740 nm [21–22,40]. In addition, modulation of the L–Z equilibrium through tuning of N-substituents has enabled the development of fluorogenic and spontaneously blinking SiR probes with finely controlled off–on behavior [11,26,28,41]. However, despite the maturity of photophysical optimization, the broader expansion of SiR chemistry is increasingly constrained by the limited accessibility of 5,6-carboxylated SiR derivatives. The carboxy groups provide a general and efficient handle for amide coupling with targeting ligands and are therefore central to the construction of SiR-based probes, including HaloTag, SNAP-tag, peptide-, and small-molecule-conjugated derivatives [18,35]. The synthetic accessibility of 5,6-carboxylated SiRs has thus become a central bottleneck in the diversification and broader application of SiR chemistry.

Traditional approaches to the synthesis of 5,6-carboxylated SiRs generally rely on the prior installation of protected carboxy-containing precursors (Figure 1b), followed by cyclization to construct the SiR framework and subsequent deprotection [41–44]. In these routes, the strong electron-withdrawing effect of the carboxy substituent reduces substrate reactivity, thereby diminishing the efficiency of the key ring-closing step and often resulting in low overall yields [45]. Moreover, these methods commonly rely on strongly basic conditions, particularly the use of highly reactive *tert*-butyllithium, which further reduces their practical utility. In addition, the preparation of protected carboxy-group containing substrates is often cumbersome [46], especially in the synthesis of (hydroxymethyl)rhodamine intermediates, thereby further increasing synthetic complexity [41]. To overcome these limitations, Butkevich developed an improved strategy based on brominated precursors [45]. Owing to the weaker electron-withdrawing effect of bromine relative to carboxy groups, these precursors exhibit higher reactivity and enable the construction of brominated SiR scaffolds in improved yields (60–70%) under milder conditions. Subsequent Pd-catalyzed carbonylation using in situ-generated CO then furnished the corresponding carboxylated SiRs in 50–90% yields. Although this approach significantly improved synthetic efficiency, it still relies on specialized setups for CO generation and expensive palladium catalysts, thereby increasing operational complexity, safety concerns, and overall cost.

Herein, inspired by the limitations of existing methods, we report a direct CO_2_-mediated carboxylation strategy for the efficient synthesis of 5,6-carboxylated SiR derivatives from brominated SiR precursors (Figure 1c). By introducing the classical carboxylation of metallated aryl halides with CO_2_ into SiR chemistry, this method enables the efficient conversion of brominated SiRs into the corresponding carboxylated products using *n-*BuLi and readily available CO_2_ under standard Schlenk conditions. The target products are obtained in 60–93% yields under relatively mild conditions. Compared with previous approaches, this strategy avoids the need for specialized apparatus for in situ CO generation as well as expensive palladium catalysts, thereby significantly reducing experimental complexity and synthetic cost. Importantly, the resulting carboxylated SiR derivatives can be directly used in subsequent amidation reactions with targeting ligands without column chromatography, facilitating the rapid construction of high-performance targeted fluorescent probes. Overall, this strategy provides a straightforward, efficient, and practical synthetic route for the functional derivatization of SiR dyes and the development of targeted probes.

## Results and Discussion

### Synthesis of carboxy-substituted silicon-rhodamine

Inspired by Butkevich’s work [45], we envisioned that carboxylated SiRs might be accessed through brominated SiR intermediates, thereby avoiding the traditional multistep synthesis from protected carboxylated precursors. Butkevich convincingly demonstrated the generality of Pd-catalyzed carbonylation conditions employing ex situ CO for the conversion of bromo-oxorhodamines, carbo-rhodamines, and silicon-rhodamines into their corresponding carboxylated forms. However, the operational complexity of these conditions may limit their practical utility. In particular, the generation and handling of CO remain major concerns because of its toxicity and limited accessibility in standard synthetic laboratories. To address this limitation, we explored a CO-free carboxylation strategy using CO_2_ as an inexpensive, readily available, and non-toxic C1 source. In principle, CO_2_ can serve as a carbonyl source through nucleophilic addition followed by protonation to furnish the corresponding carboxylic acid.

**Figure 2:**
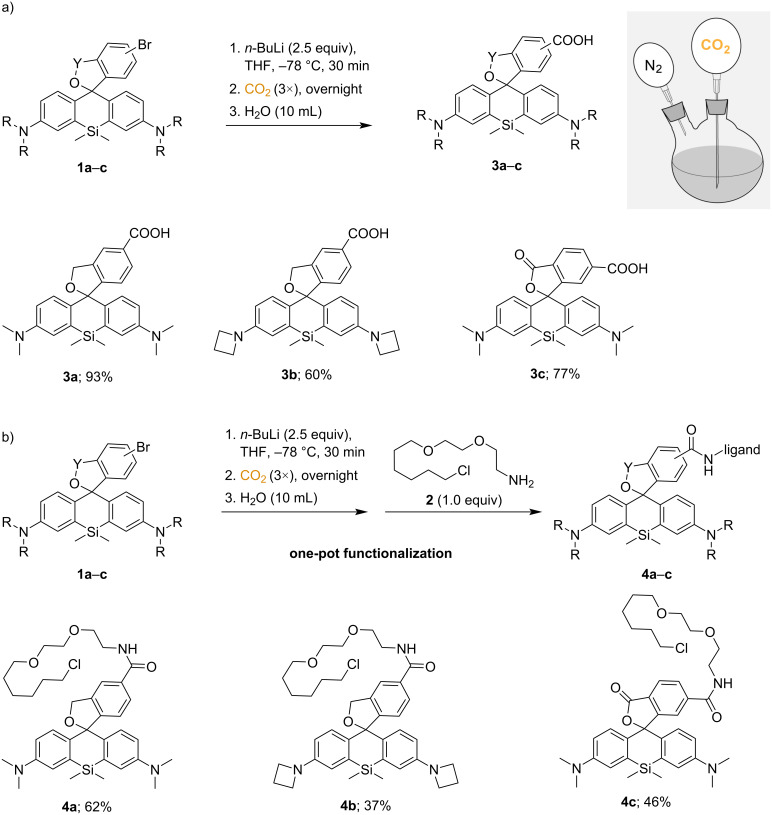
(a) Direct carboxylation of brominated silicon-rhodamines **1a**–**c** under *n-*BuLi/CO_2_. (b) Strategy for the one-pot functionalization of brominated silicon-rhodamines **1a**–**c** to construct SiR–halo fluorescent probes **4a**–**c**.

On the basis of this design (Figure 2a), brominated SiR precursors **1a**–**c** were treated with 2.5 equiv of *n-*BuLi in anhydrous THF at −78 °C under a nitrogen atmosphere to generate the corresponding aryllithium intermediates, followed by introduction of CO_2_ via a balloon. The reaction mixture was then allowed to warm to room temperature and stirred overnight to ensure complete trapping of the aryllithium intermediates by CO_2_. After aqueous quenching and extraction, the crude products were directly subjected to HPLC analysis.

**Figure 3:**
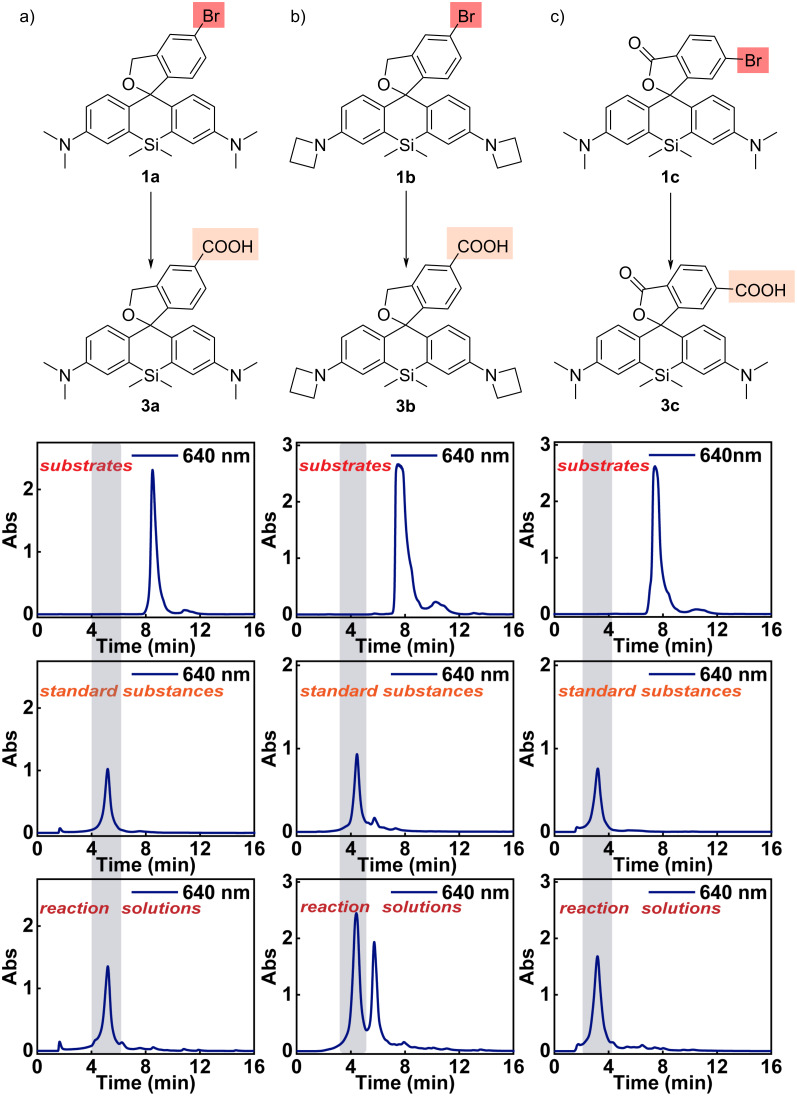
(a–c) Conversion of three bromo-SiR precursors **1a**–**c** in the CO_2_ carboxylation reaction and their corresponding HPLC results.

To evaluate the generality of this strategy, three brominated SiR precursors **1a**–**c**, all accessible by reported procedures, were selected as model substrates (Figure 3). Direct HPLC analysis of the crude reaction mixtures at 640 nm revealed clear separation between substrates and products. The retention times of **1a**, **1b**, and **1c** were 8.5, 7.5, and 7.4 min, respectively, whereas those of the corresponding carboxylated products **3a**, **3b**, and **3c** were 5.2, 4.4, and 3.2 min, allowing unambiguous distinction between starting materials and products. On the basis of peak integration, products **3a**, **3b**, and **3c** were obtained in 93%, 60%, and 77% yields, respectively. Compared with previously reported routes, this protocol provides a simpler and safer approach to carboxylated SiR derivatives while delivering good to excellent yields.

### Sequential one-pot functionalization of brominated silicon-rhodamine

In the carboxylation of **1b**, a prominent side peak was observed in addition to the desired product peak (Figure 3b). NMR (Supporting Information File 1, Figure S17) and HRMS (Figure S24) analysis identified this side product as the uncarboxylated rhodamine formed by lithium–halogen exchange followed by protonation. This byproduct likely arises from protonation of the aryllithium intermediate before efficient trapping by CO_2_, possibly due to trace moisture or other proton sources in the reaction system. Improving the CO_2_-trapping efficiency and maintaining strictly anhydrous conditions may help suppress this side reaction. Similar uncarboxylated rhodamine byproducts were also present in the reaction mixtures of the other two precursors, although in lower amounts. Importantly, these species lack a carboxy group and therefore cannot participate in subsequent amide coupling reactions. Moreover, although small amounts of these byproducts were formed, they did not significantly interfere with the subsequent reactions. Thus, the reaction mixtures could be extracted with ethyl acetate and concentrated before being directly subjected to amide coupling without chromatographic purification.

To demonstrate the utility of this strategy for probe construction, we selected three representative SiR scaffolds **1a**–**c**, which were widely used in super-resolution imaging, for direct functionalization with HaloTag ligands through a one-pot sequence (Figure 2b). After treatment of compounds **1a**–**c** with *n-*BuLi and CO_2_, the reaction mixtures were extracted with ethyl acetate and concentrated, and the resulting crude carboxylated intermediates were directly subjected to amide coupling with the HaloTag amine using HATU/DIPEA as the activation system. This strategy afforded three high-performance fluorescent probes **4a**, **4b**, and **4c**, in overall yields of 62%, 37%, and 46%, respectively. The absorption and emission spectra of **4a**, **4b**, and **4c** (Figures S1–S3 in Supporting Information File 1) showed characteristic SiR fluorescence with emission maxima around 640 nm, making them suitable for far-red fluorescence imaging. These results demonstrate that the present CO_2_-mediated strategy provides a simple, safe, and efficient route to carboxylated SiR derivatives and enables their rapid conversion into targeted SiR probes.

### Long-term live-cell super-resolution imaging using one-pot-functionalized silicon-rhodamine probes

**Figure 4:**
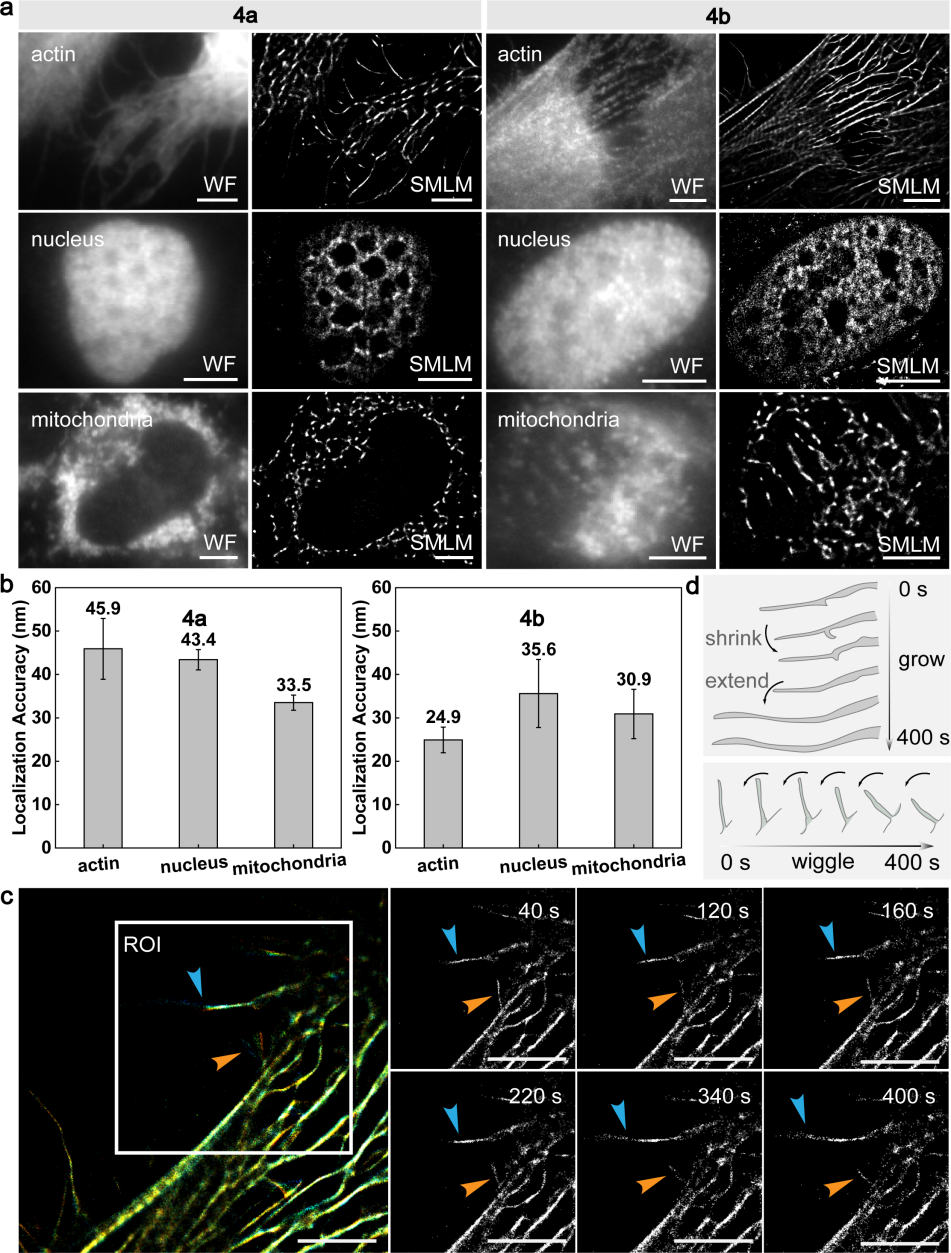
SMLM imaging of different organelles in live HeLa cells stained with **4a** and **4b** (150 nM). (a) SMLM and wide-field images of actin labeled with Lifeact-HaloTag fusion protein, nucleus labeled with H_2_B-HaloTag fusion protein, and mitochondria labeled with TOMM20-HaloTag fusion protein. (b) Average localization precisions of **4a** and **4b** for different organelles. (c) Long-term SMLM imaging of actin with **4b** in live HeLa cells. (d) Schematic diagram of filopodia growth and wiggling process in (c). Scale bars: 5.0 μm.

Encouraged by the successful one-pot functionalization of silicon-rhodamine scaffolds, we next evaluated the performance of the resulting HaloTag-targeted SiR probes in long-term super-resolution imaging. Among them, probes **4a** and **4b**, corresponding to **HMSiR-Halo** and **AzeHMSiR-Halo** originally reported by the Urano and Xu groups [30,32–34], respectively, are widely used fluorophores for single-molecule imaging because of their excellent spontaneous blinking properties. Accordingly, the two probes obtained through our one-pot strategy were first examined by single-molecule localization microscopy (SMLM) imaging (Figure 4).

Lifeact-HaloTag, H_2_B-HaloTag, and TOMM20-HaloTag fusion proteins were transiently expressed in HeLa cells to label actin filaments, the nucleus, and the mitochondrial outer membrane, respectively. Both **4a** and **4b** enabled high-quality super-resolution imaging of these distinct subcellular structures (Figure 4a), with localization precisions ranging from 24 to 46 nm (Figure 4b). In the reconstructed SMLM images, both probes clearly resolved microfilament structures with high localization precision. Probe **4b** exhibited a localization precision of 24.9 nm due to its high brightness, superior to that of **4a** (45.9 nm). In addition, filopodia displayed a width of 69.4 nm in the SMLM images, whereas the corresponding width measured by wide-field (WF) imaging was 348.8 nm (Figure S4 in Supporting Information File 1), further demonstrating the excellent performance of these probes in super-resolution imaging.

On the basis of its superior localization precision, compound **4b** was further used to investigate microfilament dynamics by long-term live-cell SMLM imaging (Figure 4c). Segmental reconstruction using continuously acquired image sequences enabled real-time tracking of filopodial growth and wiggling dynamics (Figure 4d). During the first 0–160 s, the filopodium indicated by the blue arrow underwent slight retraction, followed by gradual extension. Its length increased from 6.03 μm to 11.16 μm over the subsequent approximately 240 s (Figure S5 in Supporting Information File 1). In addition, the filopodium labeled by the orange arrow exhibited a pronounced wiggling process, with the angle between the filopodium and the cell membrane edge increasing from 50° to 95° (Figure S6 in Supporting Information File 1). This demonstrates that compound **4b** is suitable for long-term dynamic super-resolution imaging.

**Figure 5:**
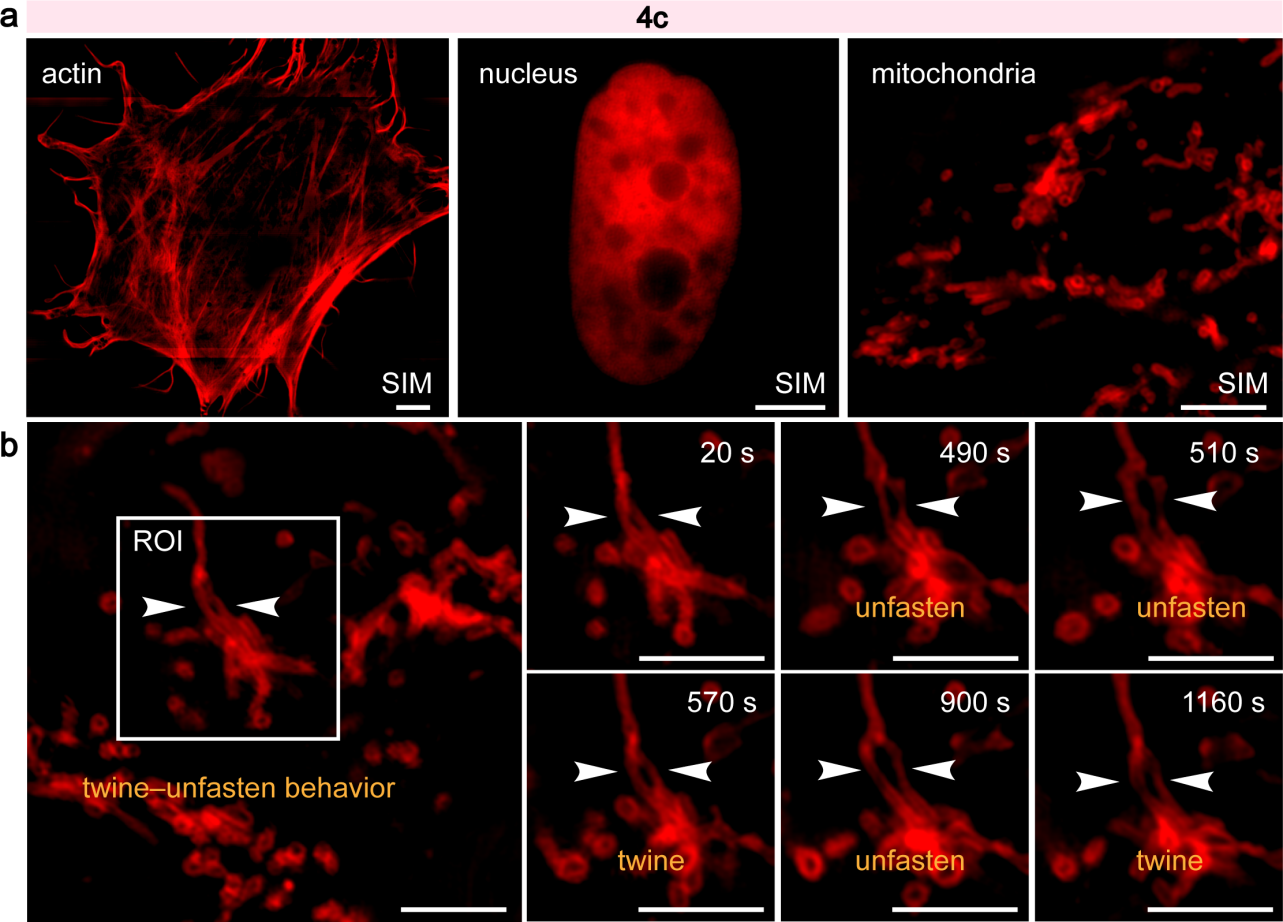
SIM imaging of different organelles in live HeLa cells stained with **4c** (200 nM). (a) SIM images of actin labeled with Lifeact-HaloTag fusion protein, nucleus labeled with H_2_B-HaloTag fusion protein, and mitochondria labeled with TOMM20-HaloTag fusion protein. (b) Long-term SIM imaging of mitochondria with **4c** in live HeLa cells. Scale bars: 5.0 μm.

Compound **4c** is a practical fluorogenic HaloTag probe for live-cell imaging. In previous studies, HaloTag-compatible rhodamine probes have been used to visualize a variety of intracellular proteins, including histone H_2_B, Sox2, vimentin, clathrin, and ensconsin [15–16,39], underscoring the broad utility of this labeling strategy for protein-specific imaging. Subsequently, the **4c** probe obtained through our one-pot strategy was applied to SIM imaging of multiple subcellular structures, including actin filaments, nucleus, and mitochondria (Figure 5a). Long-term live-cell imaging of mitochondria over 20 min was successfully performed, revealing pronounced temporal remodeling of the mitochondrial network, including recurrent twine-unfasten events (Figure 5b). Specifically, at 20 s, two neighboring mitochondria marked by the white arrow were in a twined state. By 490 s, they gradually unfastened, followed by further separation at 510 s. At 570 s, the twined state reappeared, whereas at 900 s, a clearly unfastened state was again observed, followed by gradual re-twining. These observations demonstrate the reversible and highly dynamic nature of mitochondrial twine-unfasten behavior and highlight the utility of **4c** for long-term dynamic super-resolution imaging.

## Conclusion

In conclusion, we have developed a practical CO_2_-mediated strategy for the synthesis of 5,6-carboxylated silicon-rhodamine (SiR) derivatives from brominated SiR precursors via lithium–halogen exchange and direct carboxylation. This method enables efficient access to carboxylated SiRs under mild conditions while avoiding the use of *t*-BuLi, toxic CO, and expensive palladium catalysts. The crude carboxylation mixtures can be directly advanced to amide coupling without chromatographic purification, allowing streamlined one-pot construction of functionalized SiR probes. Using this approach, several high-performance probes were readily prepared and successfully applied to long-term live-cell super-resolution imaging. Overall, this work improves the synthetic accessibility of functionalized SiR dyes and provides a concise and versatile platform for the rapid development of targeted super-resolution probes.

## Supporting Information

File 1 Experimental details and characterization of compounds by NMR and ESIMS.

## Data Availability

All data that supports the findings of this study is available in the published article and/or the supporting information of this article.
