# Supplementary material for: A practical CO2-mediated synthesis of 5,6-carboxylated silicon-rhodamines for targeted probe development
Source: Beilstein J Org Chem. 2026 Jun 10;22:915–24. doi: 10.3762/bjoc.22.72 (PMC13267498; doi:10.3762/bjoc.22.72)
Supplement: File 1 — Experimental details and characterization of compounds by NMR and ESIMS. [file Beilstein_J_Org_Chem-22-915-s001.pdf]

BEILSTEIN JOURNAL OF ORGANIC CHEMISTRY

Supporting Information

for

A practical CO_2-mediated synthesis of 5,6-carboxylated silicon-rhodamines for targeted probe development

Dongjie Hou, Shaowei Wu, Ning Xu, Pengjun Bao, Wenhao Jia, Qinglong Qiao and Zhaochao Xu

Beilstein J. Org. Chem. **2026,** *22,* 915–924. doi:10.3762/bjoc.22.72

Experimental details and characterization of compounds by NMR and ESIMS

License and Terms: This is a supporting information file under the terms of the Creative Commons Attribution License (https://creativecommons.org/licenses/by/4.0). Please note that the reuse, redistribution and reproduction in particular requires that the author(s) and source are credited and that individual graphics may be subject to special legal provisions.
The license is subject to the *Beilstein Journal of Organic Chemistry* terms and conditions: (https://www.beilstein-journals.org/bjoc/terms)

Table of contents

1. Experimental details S1
    1.1 Materials and instrumentation S1
    1.2 Cell culture and transfection S1
        1.2.1 Cell culture S1
        1.2.2 Cell transfection S1
    1.3 Staining and imaging S2
    1.4 Localization precision analysis S2
    1.5 UV–vis absorption spectra and fluorescence spectra S2
    1.6 Imaging details S3
2. Synthesis methods S4
    2.1 Preparation of the reaction precursors **1a**–**c** and **2** S4
    2.2 Synthetic procedure for carboxy-SiR **3a**–**c** S5
        2.2.1 Synthesis of **3a** S5
        2.2.2 Synthesis of **3b** S5
        2.2.3 Synthesis of **3c** S6
        2.2.4 CO_2 introduction procedure S7
    2.3 Sequential one-pot functionalization via synthetic protocol **4a**–**c** S7
        2.3.1 Synthesis of **4a** S7
        2.3.2 Synthesis of **4b** S8
        2.3.3 Synthesis of **4c** S9
3. Compound characterizations S10
4. References S17

1. Experimental details

1.1 Materials and instrumentation

Unless explicitly stated, all reagents were purchased from commercial suppliers (Sigma Aldrich, J&K, Innochem, and Aladdin) and used without further purification. Solvents (dimethyl sulfoxide (DMSO), dimethylformamide (DMF), methanol) were purchased from J&K and used without further treatment or distillation. Silica gel (200–300 mesh) and aluminum oxide basic (200–300 mesh) were purchased from Innochem.

^{1}H NMR and ^{13}C NMR spectra were recorded on a Bruker 400M spectrometer or Bruker 700M spectrometer with TMS as an internal standard. Chemical shifts were given in ppm and coupling constants (J) in Hz. High-resolution mass spectrometry data were obtained with an HP1100LC/MSD mass spectrometer and an LC/Q-TOF MS spectrometer. UV–vis absorption spectra were collected on an Agilent Cary 60 UV–vis spectrophotometer. Fluorescence measurements were performed on an Agilent CARY Eclipse fluorescence spectrophotometer.

Super-resolution images were performed by using a Nikon N-STORM/SIM 5.0 Super-Resolution Microscope System with a motorized inverted microscope ECLIPSE Ti2-E, a 100 × / NA 1.49 oil immersion TIRF objective lens (CFI HP), LU-NV series laser unit (405 nm, 488 nm, 561 nm, 647 nm), and an ORCA-Flash 4.0 SCMOS camera (Hamamatsu Photonics K.K.).

1.2 Cell culture and transfection

1.2.1 Cell culture

HeLa cells were purchased from the Cell Bank of Type Culture Collection of the Chinese Academy of Sciences. Cells were maintained in Dulbecco's modified Eagle's medium (DMEM, Gibco) supplemented with 10% fetal bovine serum (FBS, Hyclone), which were cultured in a humidified atmosphere of 5% CO_2/95% air at 37 °C. Before the imaging experiments, cells were seeded on a glass bottom cell culture dish (Nest, polystyrene, Ø 15 mm) for 1–2 days to reach 50–80% confluency. The cells were then used for further experiments.

1.2.2 Cell transfection

In a manner similar to ref [1], Halo-H_2B, Halo-actin, and Halo-TOMM20 plasmids were purchased from Addgene and transfected into cells using Lipofectamine 2000 (Invitrogen) according to the manufacturer's protocol.

For transfection, 2 μL Lipofectamine 2000 (Invitrogen) and the appropriate plasmid were first diluted in 20 μL DMEM (Dulbecco's Modified Eagle Medium), respectively. After incubation for 5 min, the diluted plasmid in 20 μL DMEM was added to the diluted Lipofectamine 2000 solution and gently mixed. The resulting mixture was further

incubated for 10 min and then added to the cell-culture dish in 1 mL DMEM. The final plasmid concentration was adjusted to 1600 ng/mL. After incubating for 4 h at 37 °C, the culture medium was changed from DMEM to DMEM with 10% FBS. The transfected cells were cultured for an additional 24–48 h prior to super-resolution imaging experiments.

1.3 Staining and imaging

Live HeLa cells were incubated with probes (150 nM or 200 nM) in DMEM for 1 h at 37 °C under 5% CO_2. Because the spirolactone-based probes exhibited low background fluorescence in the unbound state, cells were directly used for imaging without washing after incubation. For standard fluorescence imaging, excitation was at 640 nm and emission was collected at 650–750 nm. For structured illumination microscopy (SIM) and single-molecule localization microscopy (SMLM), cells were imaged immediately following the staining under the respective imaging conditions.

1.4 Localization precision analysis

The reported localization precision values (24–46 nm) represent the average of 3 to 5 independent fields of view per sample. The raw precision data for individual localized single-molecule events in each field of view were directly exported using the analysis software integrated with the Nikon N-STORM/SIM 5.0 system. The final reported range was calculated by averaging these datasets.

1.5 UV–vis absorption spectra and fluorescence spectra

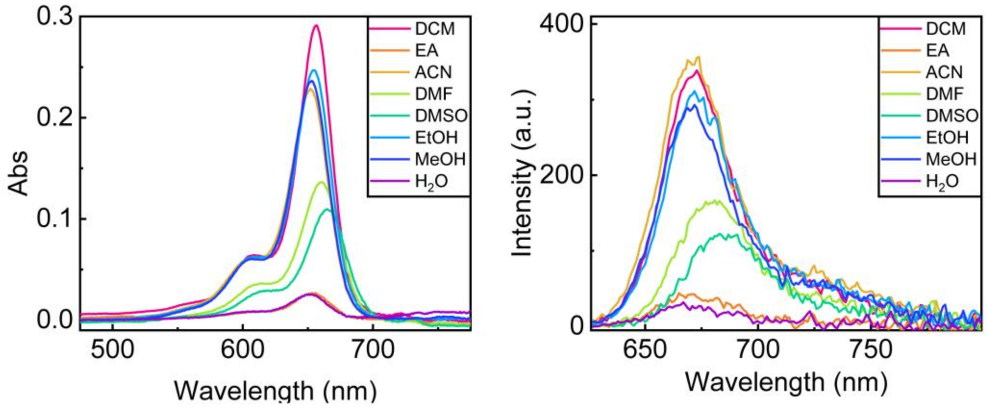

Figure S1 UV–vis absorption and fluorescence spectra of **4a** in different solvents (with 1‰ CF_3COOH).

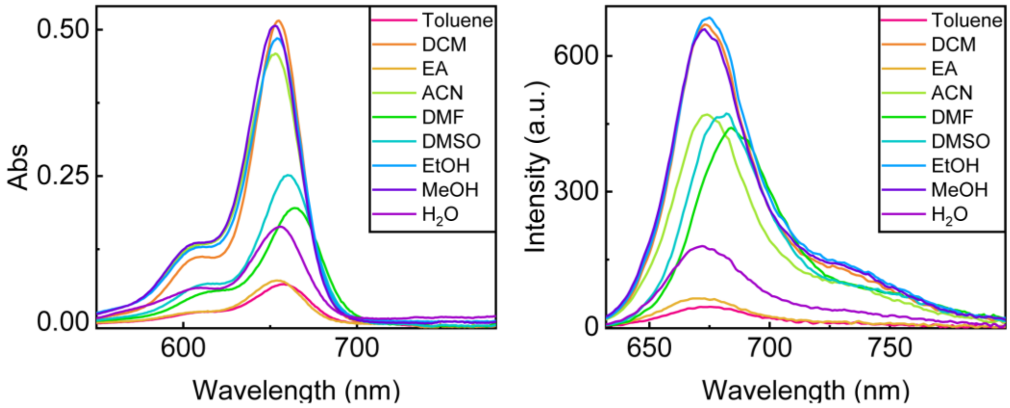

Figure S2 UV–vis absorption and fluorescence spectra of **4b** in different solvents (with 1‰ CF_3COOH).

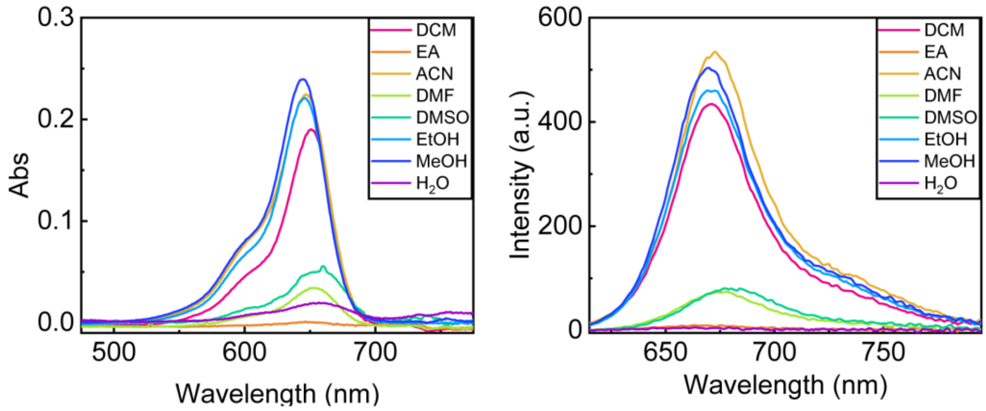

Figure S3 UV–vis absorption and fluorescence spectra of **4c** in different solvents (with 1‰ CF_3COOH).

1.6 Imaging details

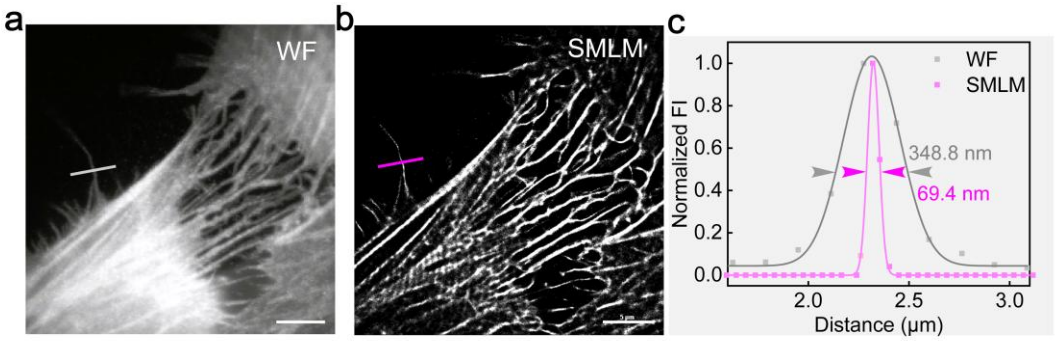

Figure S4 (a, b) Wide-field and SMLM images of actin labeled with compound **4b** in live HeLa cells. (c) Intensity profiles along the filopodia indicated by the white line in (a) and the pink lines in (b). Scale bars: 5.0 μm.

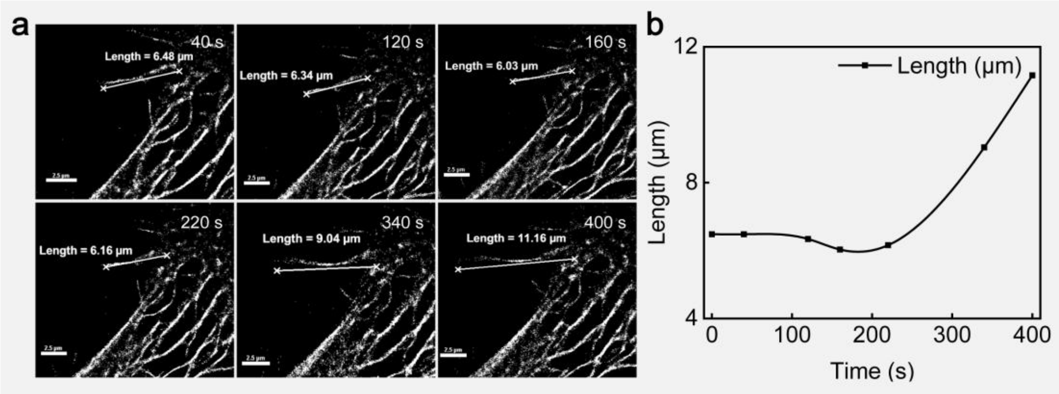

Figure S5 (a) Filopodia length at different time points in live HeLa cells labeled with compound **4b**. (b) Quantitative analysis of filopodia length in (a). Scale bars: 2.5 μm.

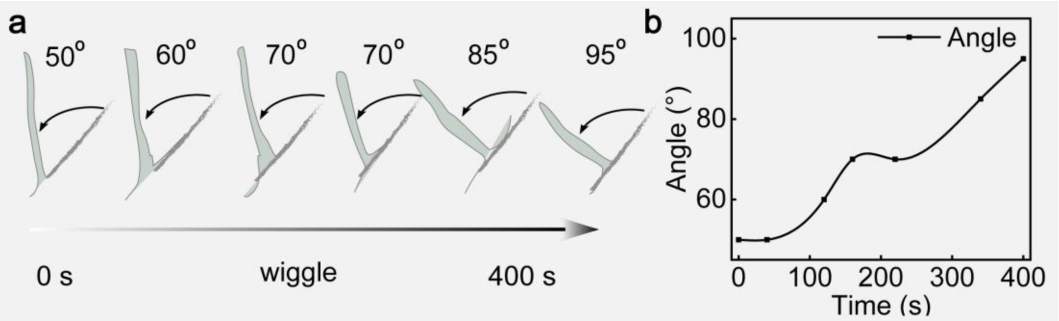

Figure S6 (a) Wiggling angles of filopodia at different time points in live HeLa cells labeled with compound **4b**. (b) Quantitative analysis of filopodia wiggling angles in (a).

2. Synthesis methods

2.1 Preparation of the reaction precursors 1a–c and 2

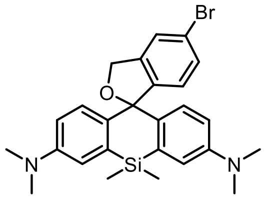

1a

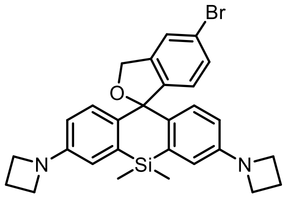

1b

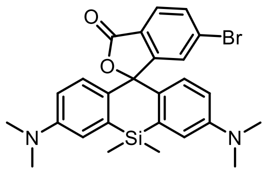

1c

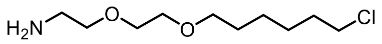

2

Synthetic procedures of **1a** and **1b** from [2]. Synthetic procedure of **1c** from [3]. Synthetic procedure of **2** from [4].

2.2 Synthetic procedure for carboxy-SiR 3a–c

2.2.1 Synthesis of 3a

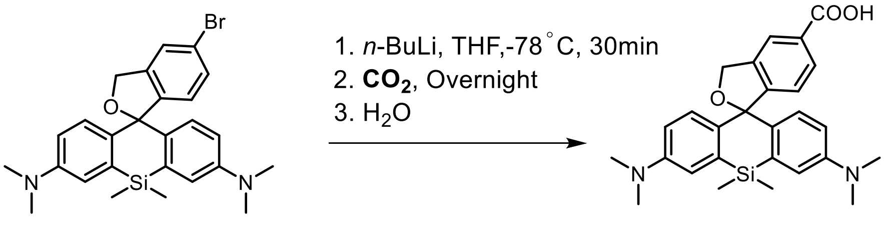

1a **3a**

In a dry Schlenk flask, **1a** (100 mg, 0.20 mmol) was charged, sealed, and evacuated/backfilled with nitrogen (3×). Anhydrous THF (10 mL) was added, and the mixture was cooled to −78 °C. *n*-Butyllithium (2.5 M in THF, 0.2 mL) was added dropwise, and the reaction mixture was stirred at −78 °C for 30 min. CO_2 was bubbled through the solution (3×), and the mixture was allowed to warm to room temperature and stirred for 10 h. The reaction was quenched with water (10 mL), and the aqueous phase was extracted with ethyl acetate (3 × 10 mL). The combined organic layers were dried over anhydrous Na_2SO_4, filtered, and concentrated. The residue was purified by silica gel chromatography (MeOH/DCM 1:10, v/v) to afford **3a** as a blue solid in 93% yield.

^{1}H NMR (400 MHz, MeOD) δ 8.01 (s, 1H), 7.84 (d, *J* = 8.0 Hz, 1H), 7.07 – 6.93 (m, 4H), 6.88 (d, *J* = 8.0 Hz, 1H), 6.68 (dd, *J* = 9.0, 2.9 Hz, 2H), 5.37 (s, 2H), 2.90 (s, 12H), 0.59 (s, 3H), 0.49 (s, 3H).

^{13}C NMR (400 MHz, MeOD) δ 153.96, 150.82, 140.00, 139.65, 135.77, 130.64, 130.28, 124.90, 124.39, 117.84, 115.98, 94.44, 74.00, 41.10.

HRMS (ESI) calcd for $C_{27}H_{31}N_2O_3Si$ $[M+H]^+$ 459.2099, found 459.2182.

2.2.2 Synthesis of 3b

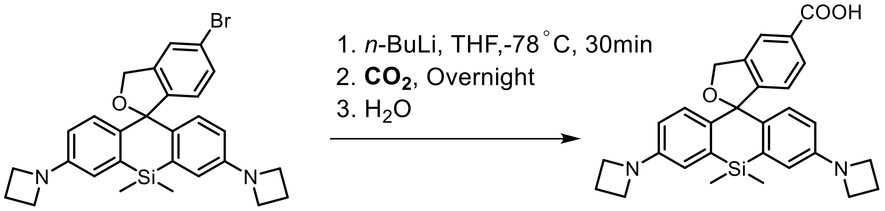

1b **3b**

In a dry Schlenk flask, **1b** (100 mg, 0.19 mmol) was charged, sealed, and evacuated/backfilled with nitrogen (3×). Anhydrous THF (10 mL) was added, and the mixture was cooled to −78 °C. *n*-Butyllithium (2.5 M in THF, 0.19 mL) was added dropwise, and the reaction mixture was stirred at −78 °C for 30 min. CO_2 was bubbled through the solution (3×), and the mixture was allowed to warm to room temperature and stirred for 10 h. The reaction was quenched with water (10 mL), and the aqueous phase was extracted with ethyl acetate (3 × 10 mL). The combined organic layers were dried over anhydrous Na_2SO_4, filtered, and concentrated. The residue was purified by silica gel chromatography (MeOH/DCM 1:10, v/v) to afford **3b** as a blue solid in 60% yield.

^{1}H NMR (400 MHz, DMSO-d_6) δ 7.92 (s, 1H), 7.75 (s, 1H), 7.01 (d, *J* = 8.2 Hz, 2H), 6.78 (s, 1H), 6.61 (s, 2H), 6.34 (d, *J* = 8.8 Hz, 2H), 5.40 (s, 2H), 3.97 – 3.57 (m, 8H), 2.28 (s, 4H), 0.56 (s, 3H), 0.43 (s, 3H).
HRMS (ESI) calcd for $C_{29}H_{31}N_2O_3Si$ $[M+H]^+$ 483.2099, found 483.2124.

During this reaction, an uncarboxylated rhodamine byproduct (compound **5**) was also isolated and characterized.

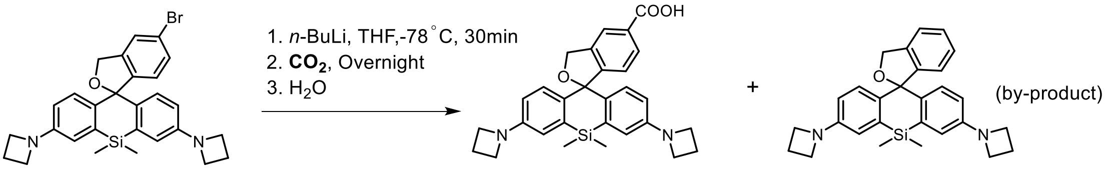

1b **3b** **5**

Compound **5**: ^{1}H NMR (400 MHz, $CDCl_3$) δ 7.29 (d, *J* = 4.6 Hz, 2H), 7.25 – 7.21 (m, 1H), 7.06 (d, *J* = 7.4 Hz, 1H), 6.95 (d, *J* = 8.6 Hz, 2H), 6.66 (d, *J* = 2.6 Hz, 2H), 6.31 (dd, *J* = 8.6, 2.6 Hz, 2H), 5.21 (s, 2H), 3.86 (t, *J* = 7.2 Hz, 8H), 2.33 (p, *J* = 7.2 Hz, 4H), 0.58 (s, 3H), 0.52 (s, 3H).
HRMS (ESI) calcd for $C_{28}H_{31}N_2OSi$ $[M+H]^+$ 439.2201, found 439.2223.

2.2.3 Synthesis of 3c

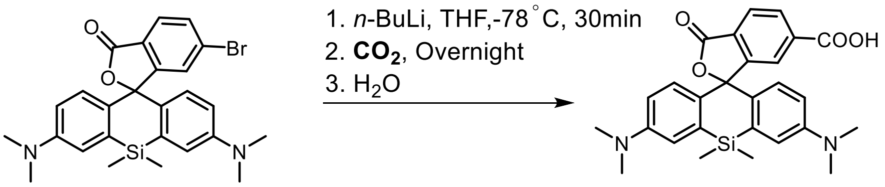

1c **3c**

In a dry Schlenk flask, **1c** (100 mg, 0.19 mmol) was charged, sealed, and evacuated/backfilled with nitrogen (3×). Anhydrous THF (10 mL) was added, and the mixture was cooled to −78 °C. *n*-Butyllithium (2.5 M in THF, 0.19 mL) was added dropwise, and the reaction mixture was stirred at −78 °C for 30 min. CO_2 was bubbled through the solution (3×), and the mixture was allowed to warm to room temperature and stirred for 10 h. The reaction was quenched with water (10 mL), and the aqueous phase was extracted with ethyl acetate (3 × 10 mL). The combined organic layers were dried over anhydrous Na_2SO_4, filtered, and concentrated. The residue was purified by silica gel chromatography (MeOH/DCM 1:10, v/v) to afford **3c** as a blue solid in 77% yield.
^{1}H NMR (400 MHz, MeOD) δ 8.22 (d, *J* = 8.0 Hz, 1H), 7.97 (d, *J* = 7.9 Hz, 1H), 7.85 (s, 1H), 7.04 (d, *J* = 2.8 Hz, 2H), 6.72 (d, *J* = 8.9 Hz, 2H), 6.62 (dd, *J* = 9.0, 2.9 Hz, 2H), 2.96 (s, 12H), 0.65 (s, 3H), 0.55 (s, 3H).
^{13}C NMR (400 MHz, MeOD) δ 173.50, 157.33, 152.49, 139.37, 133.64, 132.46, 131.41, 130.57, 127.93, 127.57, 119.24, 116.10, 50.62, 41.75, 1.55.
HRMS (ESI) calcd for $C_{27}H_{29}N_2O_4Si$ $[M+H]^+$ 473.1892, found 473.1894.

2.2.4 CO_2 introduction procedure

All carboxylation reactions were performed using high-purity (99.999%) CO_2 gas supplied from a 40 L cylinder. Approximately 0.5–1 L of CO_2 was first collected in a balloon and then introduced below the surface of the reaction mixture through a needle for approximately 3–5 min to ensure sufficient contact and reaction between CO_2 and the substrates. The CO_2-bubbling operation was typically repeated three times. For larger-scale reactions, additional CO_2 introduction cycles were applied as needed to ensure complete carboxylation.

2.3 Sequential one-pot functionalization via synthetic protocol 4a–c

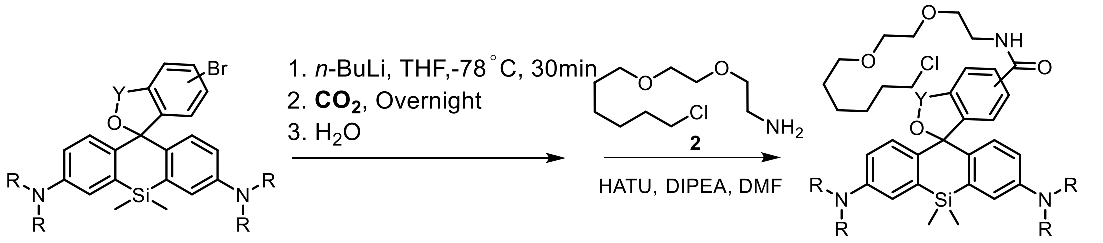

1a-c → **4a-c**

The carboxylation was performed as described above. The crude product was used directly without purification and dissolved in anhydrous DMF. Then, HATU and DIPEA were added, followed by compound **2**, and the mixture was stirred for 6 h. The solvent was removed under reduced pressure, and the residue was purified by silica gel chromatography (200–300 mesh).

2.3.1 Synthesis of 4a

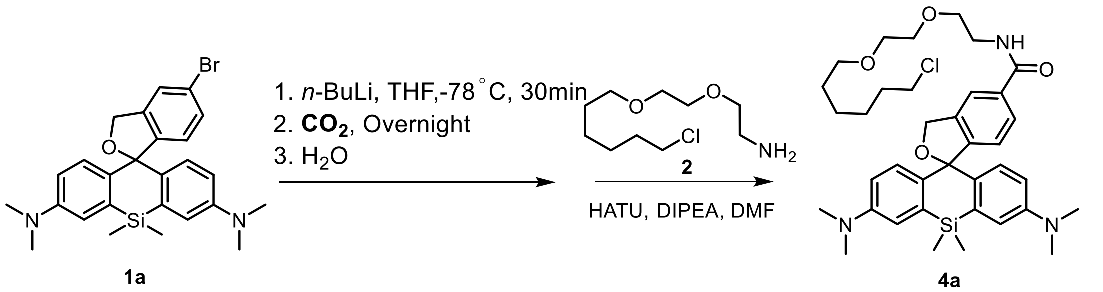

1a → **4a**

1a (50 mg, 0.10 mmol, 1 equiv) was charged in a dry Schlenk flask, sealed, and evacuated/backfilled with nitrogen (3×). Anhydrous THF (10 mL) was added, and the mixture was cooled to −78 °C. *n*-Butyllithium (2.5 M in THF, 0.1 mL, 2.5 equiv) was added dropwise, and the reaction mixture was stirred at −78 °C for 30 min. CO_2 was bubbled through (3×), then the mixture was warmed to room temperature and stirred for 10 h. After quenching with water (10 mL), the aqueous layer was extracted with ethyl acetate (3 × 10 mL), dried over Na_2SO_4, filtered, and concentrated. The crude product was dissolved in anhydrous DMF (10 mL), HATU (61 mg, 0.16 mmol, 1.6 equiv) and DIPEA (106 μL, 0.60 mmol, 6 equiv) were added, followed by **2** (22 mg, 0.10 mmol,

1.0 equiv), and the mixture was stirred for 6 h. The solvent was removed, and the residue purified by silica gel chromatography (MeOH/DCM 1:20) to give **4a** as a light green solid in 62% yield.

^{1}H NMR (400 MHz, $CDCl_3$) δ 7.77 (s, 1H), 7.64 (dd, *J* = 8.0, 1.7 Hz, 1H), 7.08 (d, *J* = 7.9 Hz, 1H), 7.04 – 6.85 (m, 4H), 6.72 (s, 1H), 6.60 (dd, *J* = 8.9, 2.9 Hz, 2H), 5.26 (s, 2H), 3.67 (dd, *J* = 10.6, 3.8 Hz, 6H), 3.59 (dd, *J* = 5.7, 3.0 Hz, 2H), 3.47 (q, *J* = 6.7 Hz, 4H), 2.95 (s, 12H), 1.75 – 1.69 (m, 2H), 1.57 (d, *J* = 7.2 Hz, 2H), 1.51 – 1.44 (m, 4H), 0.62 (s, 3H), 0.54 (s, 3H).

HRMS (ESI) calcd for $C_{37}H_{51}ClN_3O_4Si$ $[M+H]^+$ 664.3332, found 664.3341.

2.3.2 Synthesis of 4b

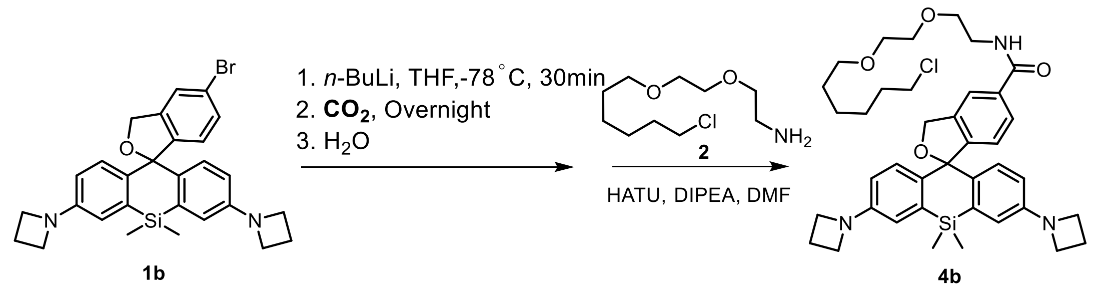

1b (30 mg, 0.05 mmol, 1 equiv) was charged in a dry Schlenk flask, sealed, and evacuated/backfilled with nitrogen (3×). Anhydrous THF (10 mL) was added, and the mixture was cooled to −78 °C. *n*-Butyllithium (2.5 M in THF, 0.06 mL, 2.5 equiv) was added dropwise, and the reaction stirred at −78 °C for 30 min. CO_2 was bubbled through (3×), then the mixture was warmed to room temperature and stirred for 10 h. After quenching with water (10 mL), the aqueous layer was extracted with ethyl acetate (3 × 10 mL), dried over Na_2SO_4, filtered, and concentrated. The crude product was dissolved in anhydrous DMF (10 mL), HATU (35 mg, 0.09 mmol, 1.6 equiv) and DIPEA (61 μL, 0.34 mmol, 6 equiv) were added, followed by **2** (13 mg, 0.05 mmol, 1 equiv), and the mixture was stirred for 6 h. The solvent was removed, and the residue purified by silica gel chromatography (MeOH/DCM 1:20) to give **4b** as a light green solid in 37% yield.

^{1}H NMR (700 MHz, $CDCl_3$) δ 7.76 (s, 1H), 7.64 (d, *J* = 7.9 Hz, 1H), 7.08 (d, *J* = 7.8 Hz, 1H), 6.90 (d, *J* = 8.6 Hz, 2H), 6.75 (s, 1H), 6.66 (d, *J* = 2.7 Hz, 2H), 6.30 (dd, *J* = 8.6, 2.6 Hz, 2H), 5.24 (s, 2H), 3.88 (t, *J* = 7.3 Hz, 8H), 3.69 – 3.65 (m, 6H), 3.59 (dd, *J* = 5.9, 3.3 Hz, 2H), 3.47 (dt, *J* = 15.9, 6.6 Hz, 4H), 2.34 (p, *J* = 7.2 Hz, 4H), 1.74 – 1.71 (m, 2H), 1.58 (d, *J* = 7.4 Hz, 2H), 1.43 – 1.38 (m, 4H), 0.58 (s, 3H), 0.52 (s, 3H).

^{13}C NMR (700 MHz, $CDCl_3$) δ 167.46, 150.41, 149.74, 140.35, 138.35, 135.39, 134.01, 128.27, 126.19, 124.63, 120.40, 115.50, 112.47, 92.62, 72.03, 71.27, 70.28, 70.02, 69.82, 52.42, 45.01, 39.76, 32.49, 29.41, 26.63, 25.38, 16.99, 1.03.

HRMS (ESI) calcd for $C_{39}H_{51}ClN_3O_4Si$ $[M+H]^+$ 688.3332, found 688.3352.

2.3.3 Synthesis of 4c

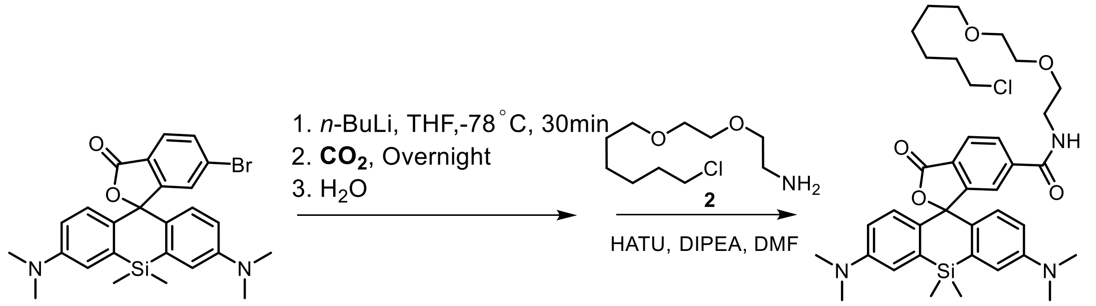

1c **4c**

1c (50 mg, 0.09 mmol, 1 equiv) was charged in a dry Schlenk flask, sealed, and evacuated/backfilled with nitrogen (3×). Anhydrous THF (10 mL) was added, and the mixture was cooled to −78 °C. *n*-Butyllithium (2.5 M in THF, 0.1 mL, 2.5 equiv) was added dropwise, and the reaction mixture was stirred at −78 °C for 30 min. CO_2 was bubbled through (3×), then the mixture was warmed to room temperature and stirred for 10 h. After quenching with water (10 mL), the aqueous layer was extracted with ethyl acetate (3 × 10 mL), dried over Na_2SO_4, filtered, and concentrated. The crude product was dissolved in anhydrous DMF (10 mL), HATU (60 mg, 0.15 mmol, 1.6 equiv) and DIPEA (103 µL, 0.59 mmol, 6 equiv) were added, followed by **2** (22 mg, 0.09 mmol, 1 equiv), and the mixture was stirred for 6 h. The solvent was removed, and the residue purified by silica gel chromatography (MeOH/DCM 1:20) to give **4c** as a light green solid in 46% yield.

^{1}H NMR (400 MHz, $CDCl_3$) δ 7.99 (d, *J* = 7.9 Hz, 1H), 7.91 (d, *J* = 8.0 Hz, 1H), 7.67 (s, 1H), 6.96 (d, *J* = 2.9 Hz, 2H), 6.78 (d, *J* = 8.9 Hz, 2H), 6.69 (s, 1H), 6.56 (dd, *J* = 9.0, 2.8 Hz, 2H), 3.63 (d, *J* = 5.1 Hz, 6H), 3.55 (d, *J* = 5.3 Hz, 2H), 3.50 (t, *J* = 6.7 Hz, 2H), 3.39 (t, *J* = 6.6 Hz, 2H), 2.97 (s, 12H), 1.80 – 1.47 (m, 8H), 0.67 (s, 3H), 0.60 (s, 3H).

^{13}C NMR (400 MHz, $CDCl_3$) δ 169.99, 166.21, 155.29, 149.37, 139.77, 136.66, 131.20, 129.92, 129.00, 128.10, 127.47, 125.81, 123.24, 116.58, 113.50, 92.00, 71.21, 70.24, 69.97, 69.55, 45.00, 40.25, 39.97, 32.47, 27.22, 26.62, 25.35, 1.02.

HRMS (ESI) calcd for $C_{37}H_{49}ClN_3O_5Si$ $[M+H]^+$ 678.3125, found 678.3132.

3. Compound characterizations

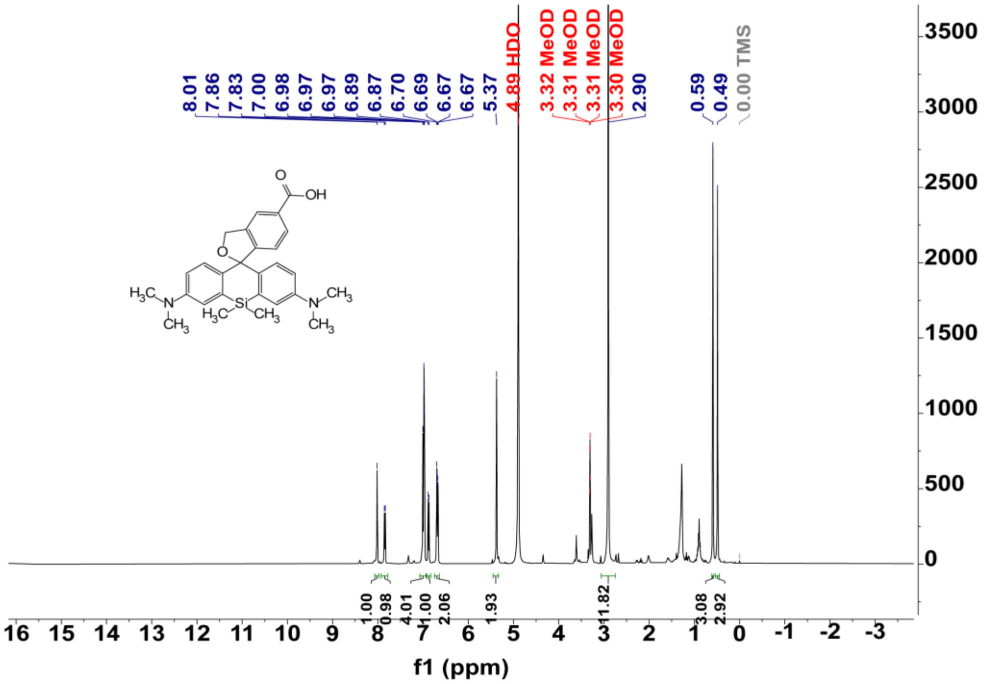

Figure S7 ^{1}H NMR spectrum of **3a** in MeOD

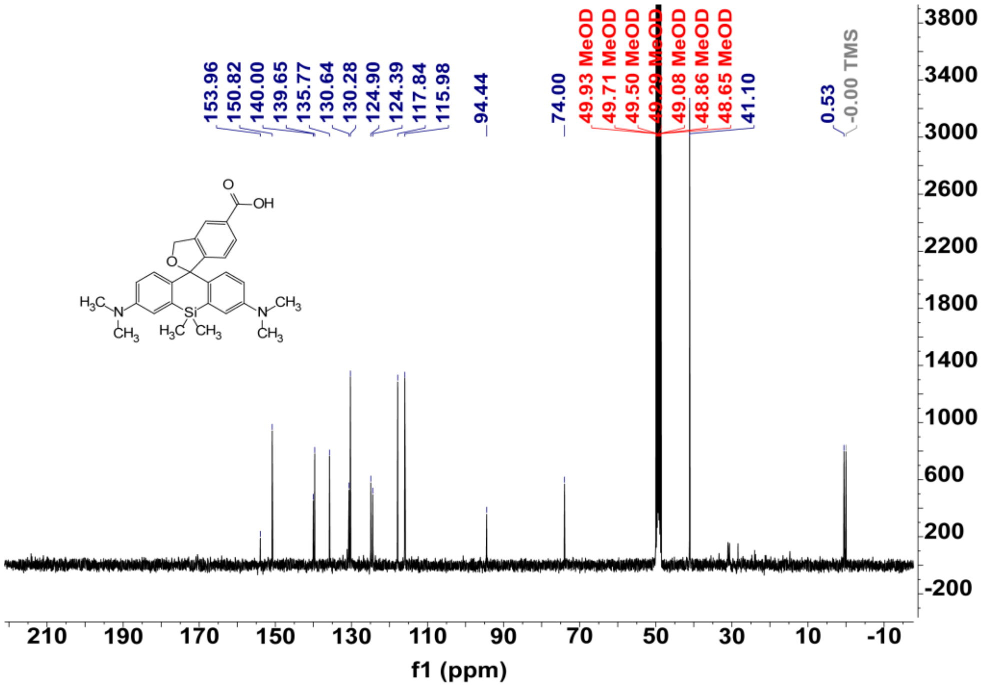

Figure S8 ^{13}C NMR spectrum of **3a** in MeOD

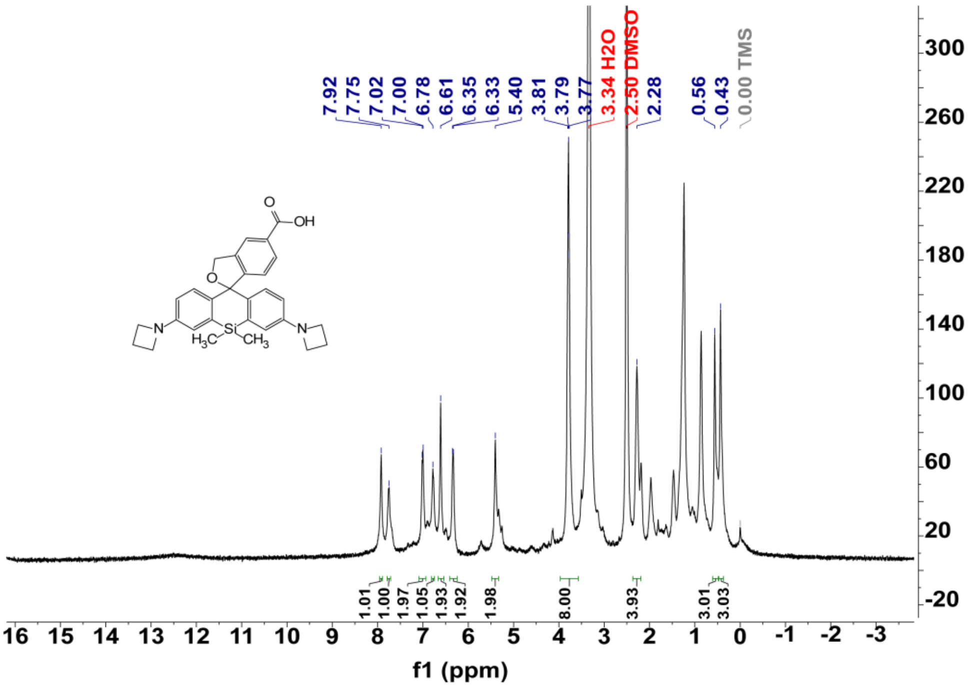

Figure S9 ^{1}H NMR spectrum of **3b** in DMSO-d_6

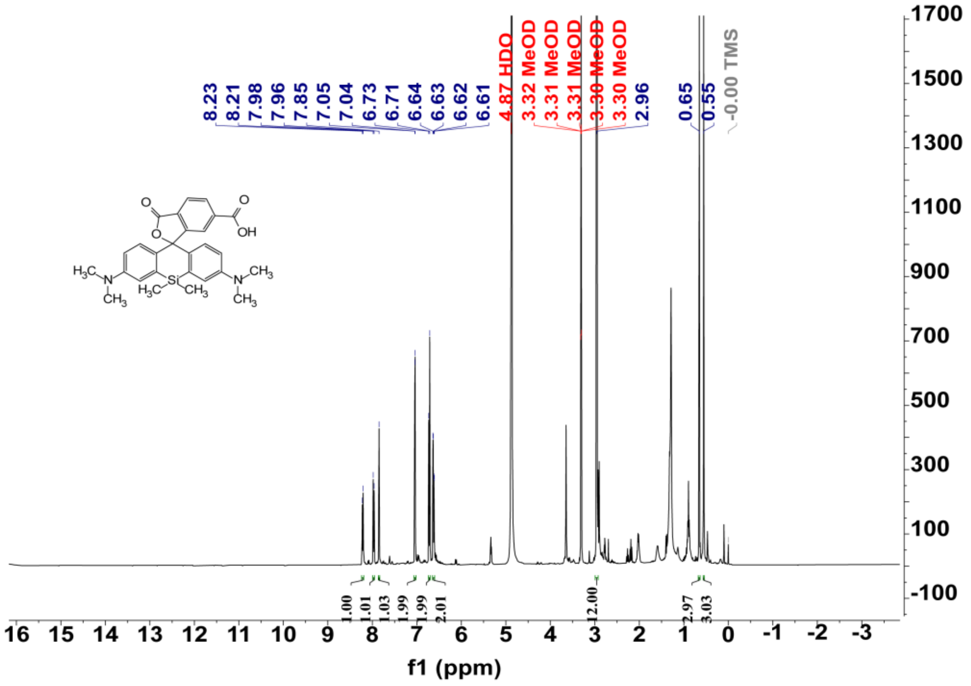

Figure S10 ^{1}H NMR spectrum of **3c** in MeOD

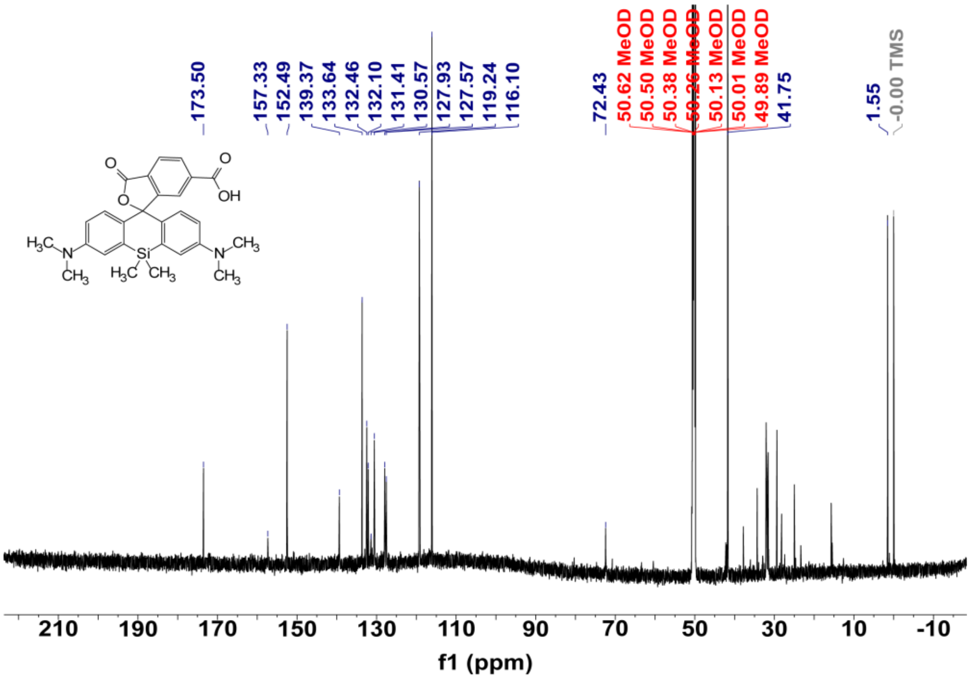

Figure S11 ^{13}C NMR spectrum of **3c** in MeOD

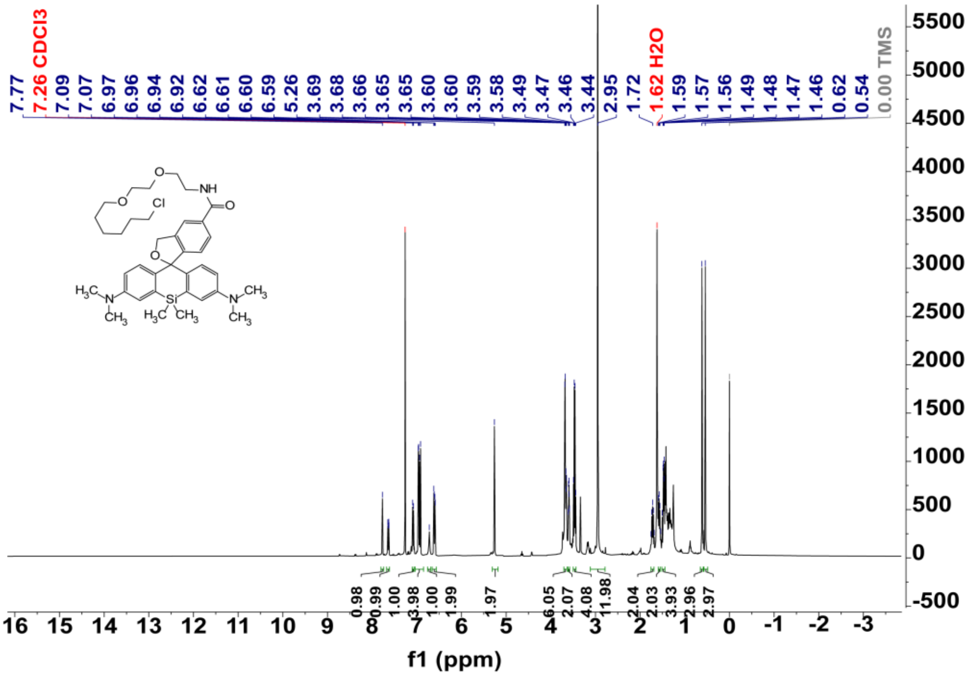

Figure S12 ^{1}H NMR spectrum of **4a** in $CDCl_3$

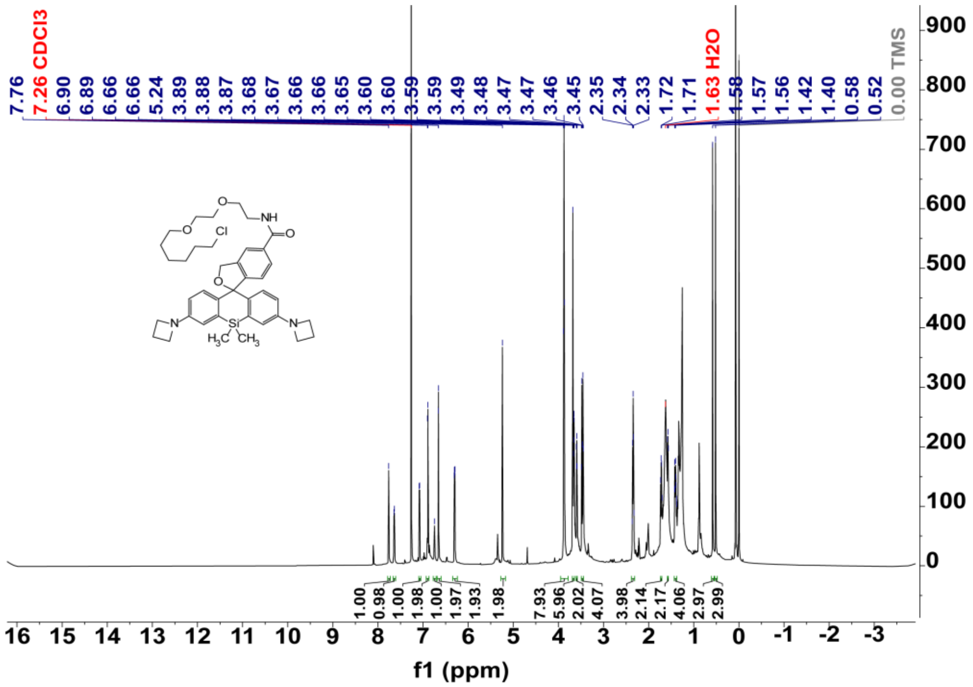

Figure S13 ^{1}H NMR spectrum of **4b** in $CDCl_3$

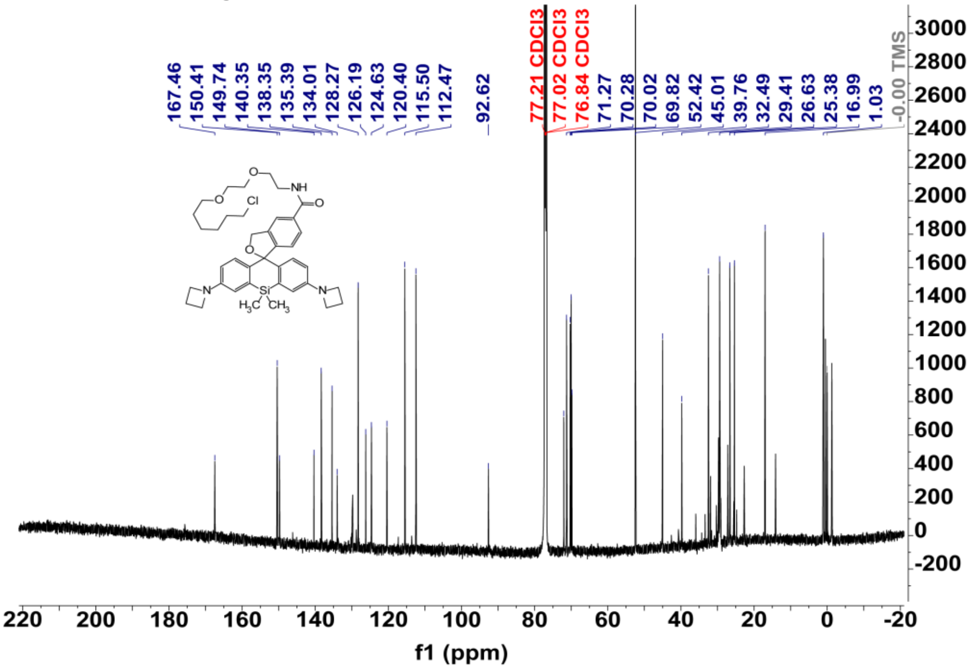

Figure S14 ^{13}C NMR spectrum of **4b** in $CDCl_3$

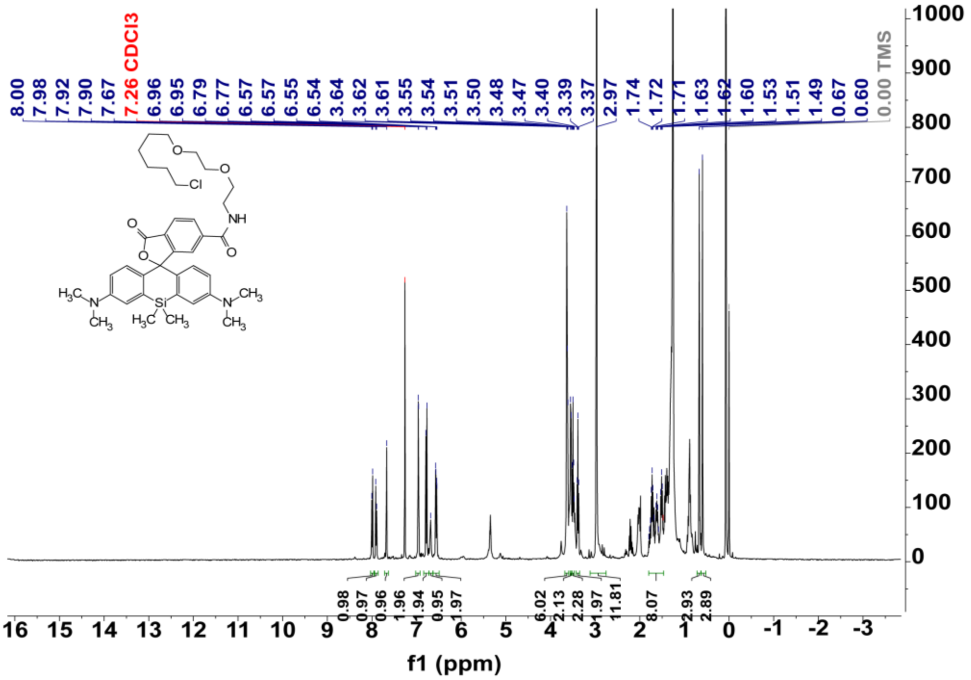

Figure S15 ^{1}H NMR spectrum of **4c** in $CDCl_3$

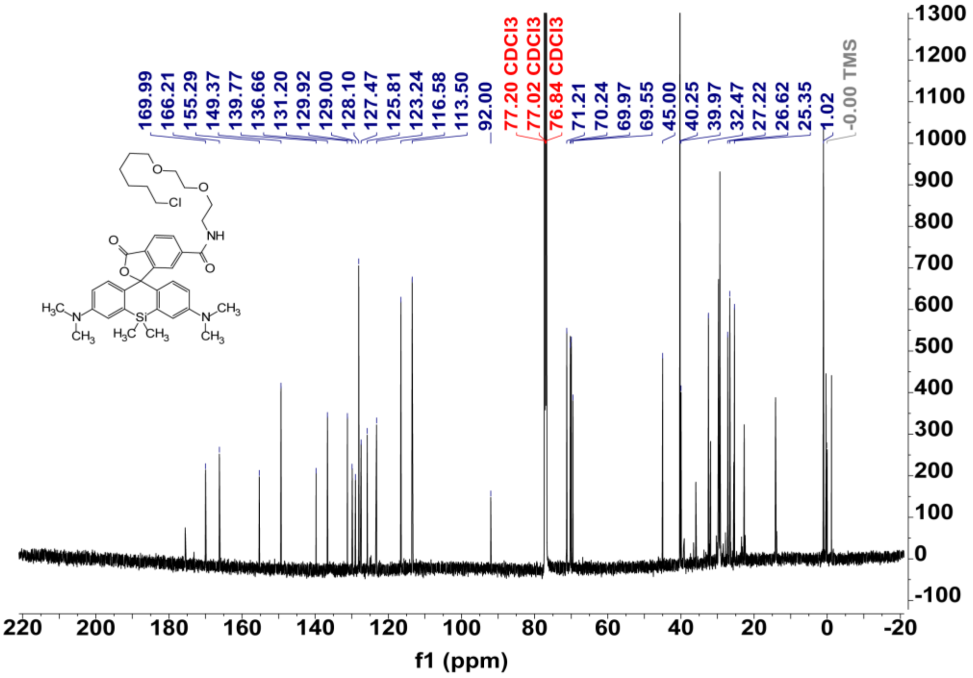

Figure S16 ^{13}C NMR spectrum of **4c** in $CDCl_3$

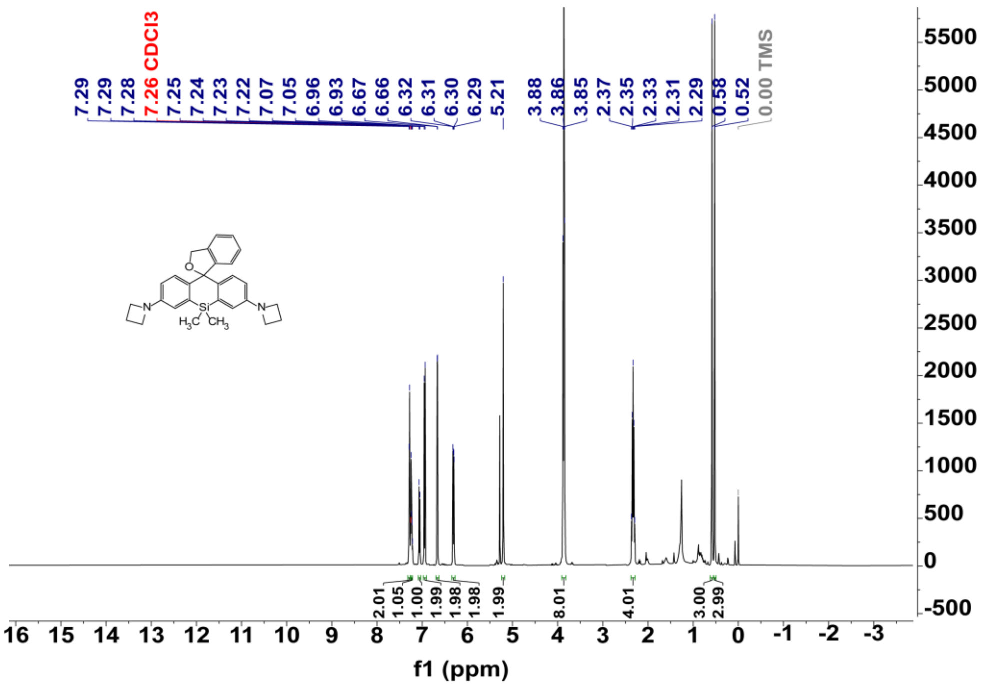

Figure S17 ^{1}H NMR spectrum of **5** in $CDCl_3$

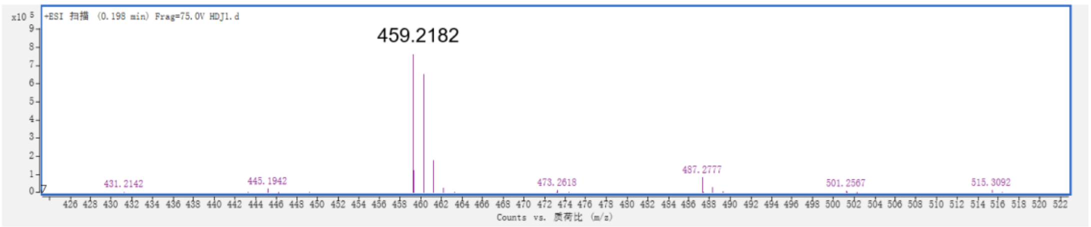

Figure S18 HRMS spectrum of **3a**

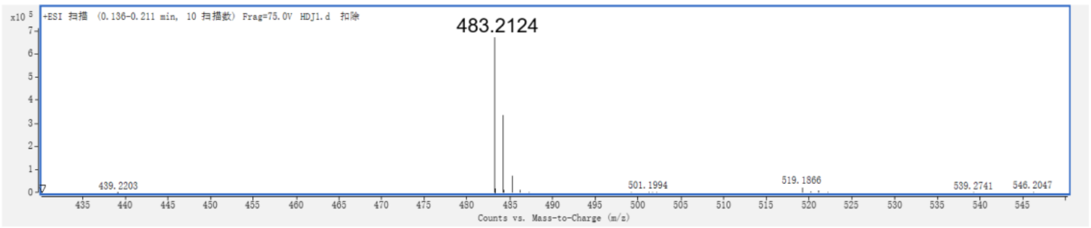

Figure S19 HRMS spectrum of **3b**

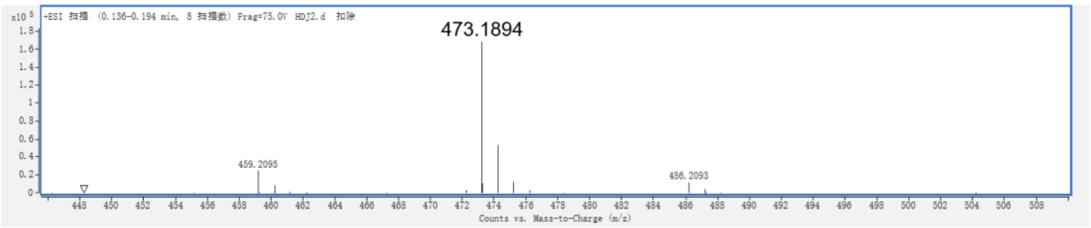

Figure S20 HRMS spectrum of **3c**

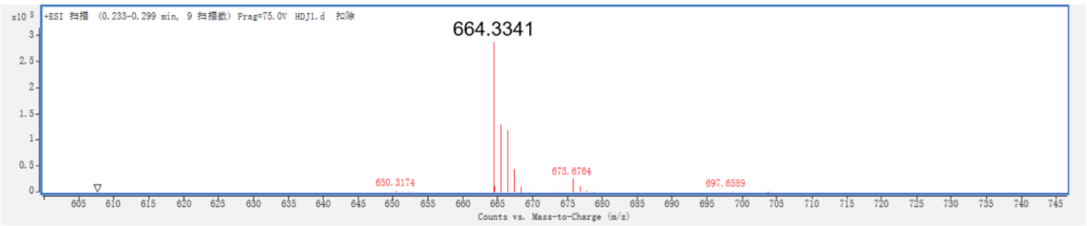

Figure S21 HRMS spectrum of **4a**

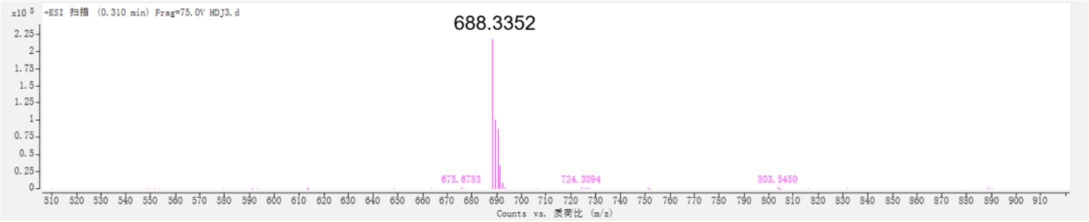

Figure S22 HRMS spectrum of **4b**

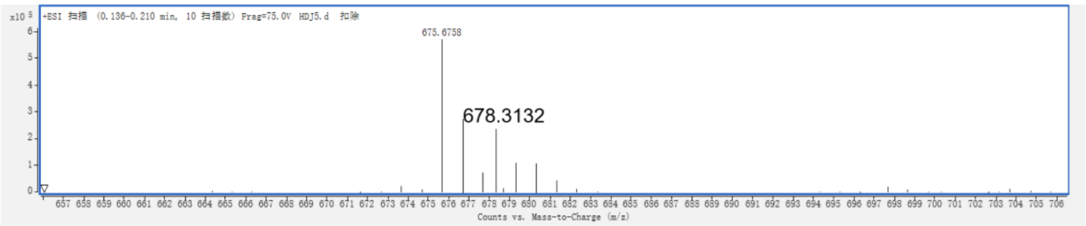

Figure S23 HRMS spectrum of **4c**

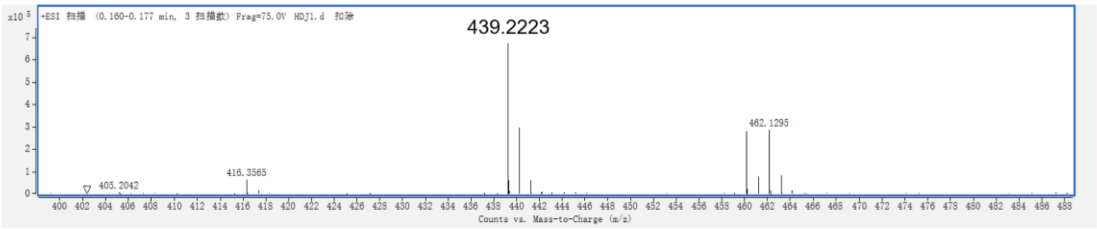

Figure S24 HRMS spectrum of **5**

4. References

1. Xu, N.; Qiao, Q.; Tao, Y.; Bao, P.; Zhang, Y.; Zhou, W.; Li, J.; Li, Z.; Xu, Z. *Sens. Actuators, B Chem.* **2024,** *398,* 134744. doi: 10.1016/j.snb.2023.134744

2. Butkevich, A. N. *Org. Lett.* **2021,** *23,* 2604-2609. doi:10.1021/acs.orglett.1c00512

3. Wang, B.; Chai, X.; Zhu, W.; Wang, T.; Wu, Q. *Chem. Commun.* **2014,** *50,* 14374-14377. doi:10.1039/c4cc06178k

4. Zhang, Y.; So, M.-k.; Loening, A. M.; Yao, H.; Gambhir, S. S.; Rao, J. *Angew. Chem., Int. Ed.* **2006,** *45,* 4936-4940. doi:10.1002/anie.200601197
